# Supplementary material for: Alginate Microsponges as a Scaffold for Delivery of a Therapeutic Peptide against Rheumatoid Arthritis
Source: Nanomaterials (Basel). 2023 Oct 5;13(19):2709. doi: 10.3390/nano13192709 (PMC10574729; doi:10.3390/nano13192709)
Supplement: Supplementary file 1 [file nanomaterials-13-02709-s001.zip › nanomaterials-2595676-supplementary.pdf]

*Supplementary Materials*

# Alginate Microsponges as a Scaffold for Delivery of a Therapeutic Peptide against Rheumatoid Arthritis

## Section S1. Determination of the NaAlg content in the microsponges

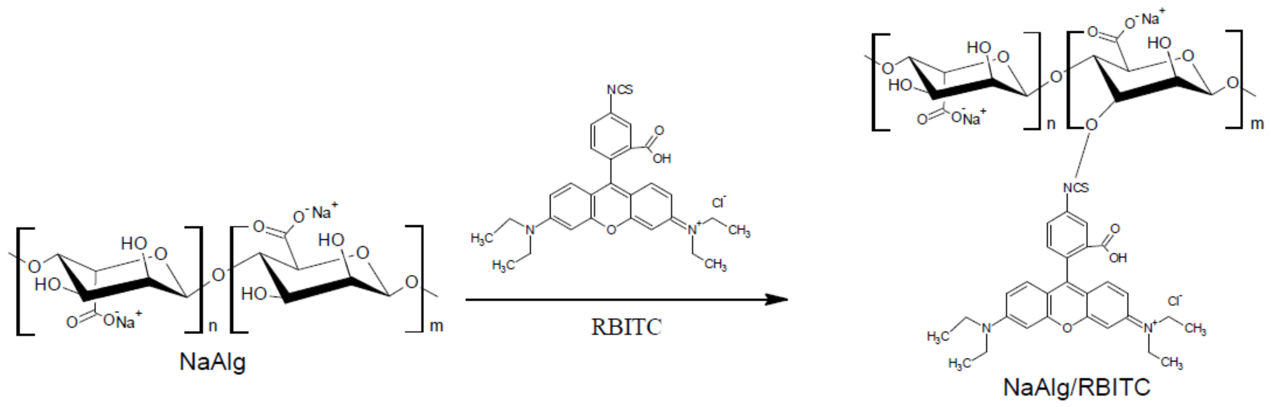


Figure S1. Reaction scheme of NaAlg with Rhodamine B Isotiocyanate.

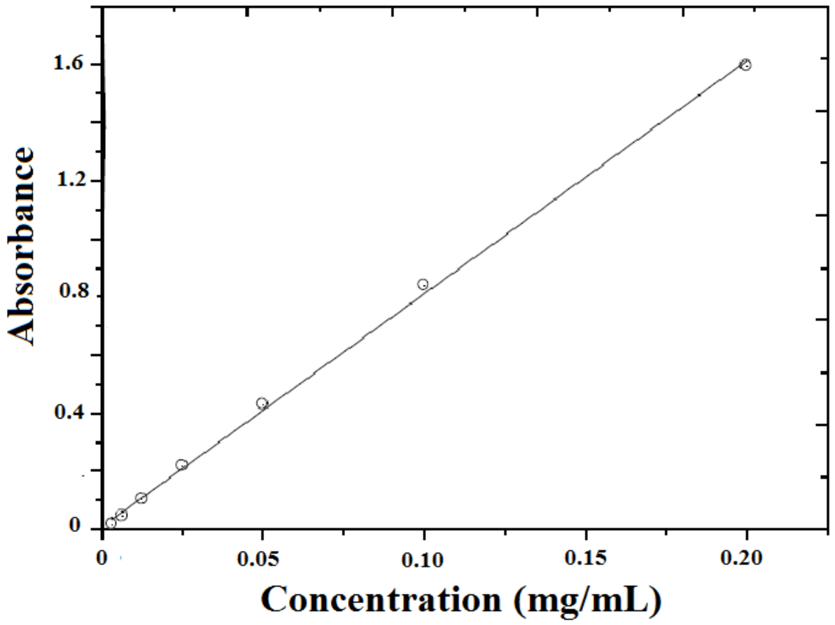


Figure S2. Calibration curve of RBITC in aqueous solution obtained by UV-Vis absorption measurements ($\lambda$=545 nm).

**Section S2. Viscosity measurements**

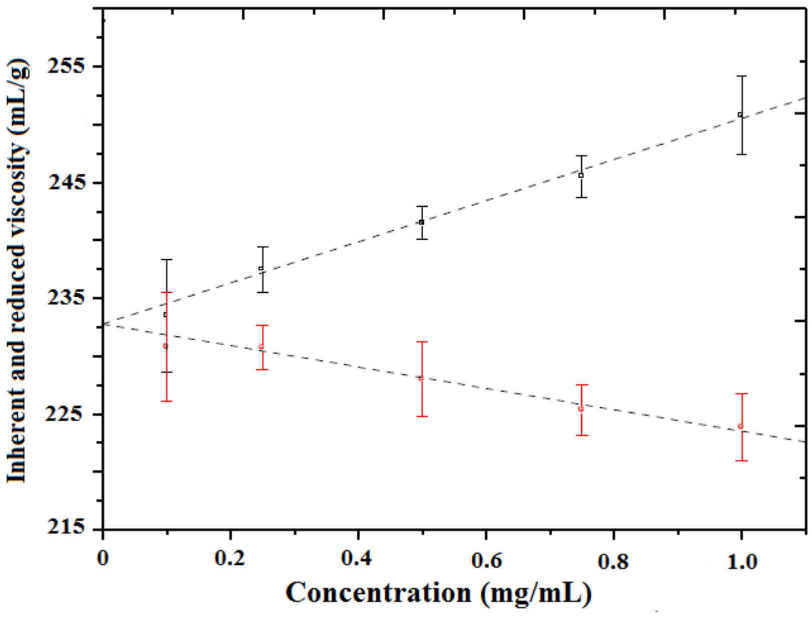


Figure S3. Viscosity measurements of NaAlg solutions in 0.1 M NaCl. Black dots: reduced viscosities; red dots: inherent viscosities. Experimental reduced and inherent viscosities (Table T1) were fitted through the Mark-Houwink-Sakurada and Huggins-Kraemer equations, respectively (dotted lines).

Table S1. Relative, specific, reduced and inherent viscosities for different concentrations of NaAlg solutions in 0.1 M NaCl (T=25°C).

| *NaAlg Concentration (mg/mL)* | $\eta_{rel}$ *(mL/g) ± SD* | $\eta_{sp}$ *(mL/g) ± SD* | $\eta_{rid}$ *(mL/g) ± SD* | $\eta_{inh}$ *(mL/g) ± SD* | *[η] (mL/g) ± SD* | *PM (kDa) ± SD* |
|---|---|---|---|---|---|---|
| 0.10 | 1.04 ±0.01 | 0.04 ± 0.01 | 233 ± 5 | 231 ± 5 | | |
| 0.25 | 1.11 ±0.01 | 0.11 ± 0.01 | 237 ± 2 | 231 ± 2 | | |
| 0.50 | 1.22 ±0.01 | 0.22 ± 0.01 | 241 ± 1 | 228 ± 3 | 233 ± 3 | 78.56 ± 0.01 |
| 0.75 | 1.35 ±0.02 | 0.35 ± 0.02 | 245 ± 2 | 225 ± 2 | | |
| 1.00 | 1.49 ±0.02 | 0.49 ± 0.02 | 251 ± 3 | 224 ± 3 | | |

**Section S3. Fluorescence of RITBC in aqueous solution and in the supernatant solution.**

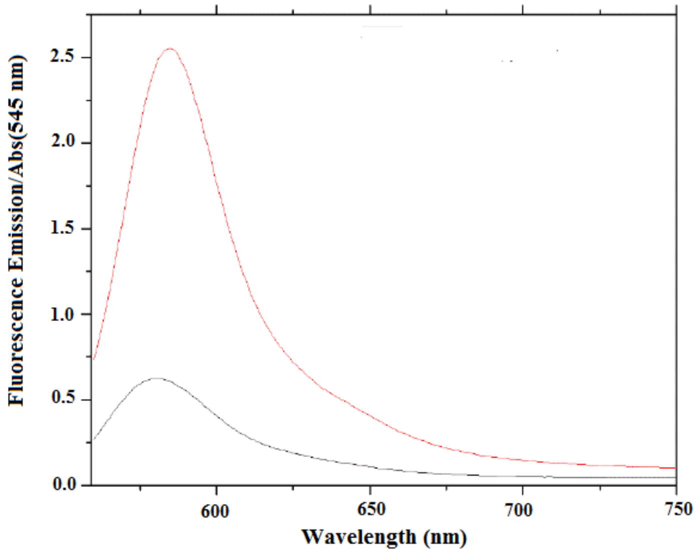


Figure S4. Emission spectra ($\lambda_{ex}$=545 nm) of RBITC/NaAlg in aqueous solution (light-blue) and as supernatant (red).

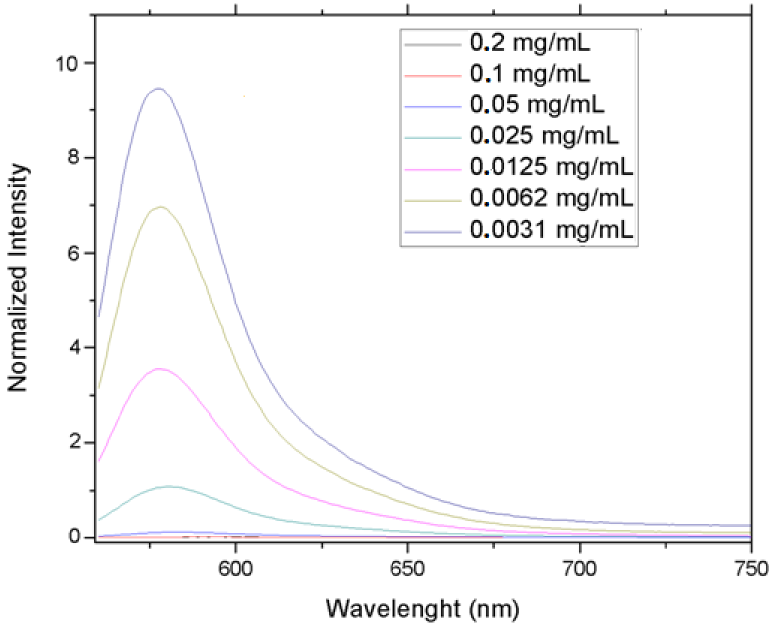


Figure S5. Fluorescence spectra of RITBC in aqueous solution at different concentrations

**Section S4. Imaging of NaAlg PMS**

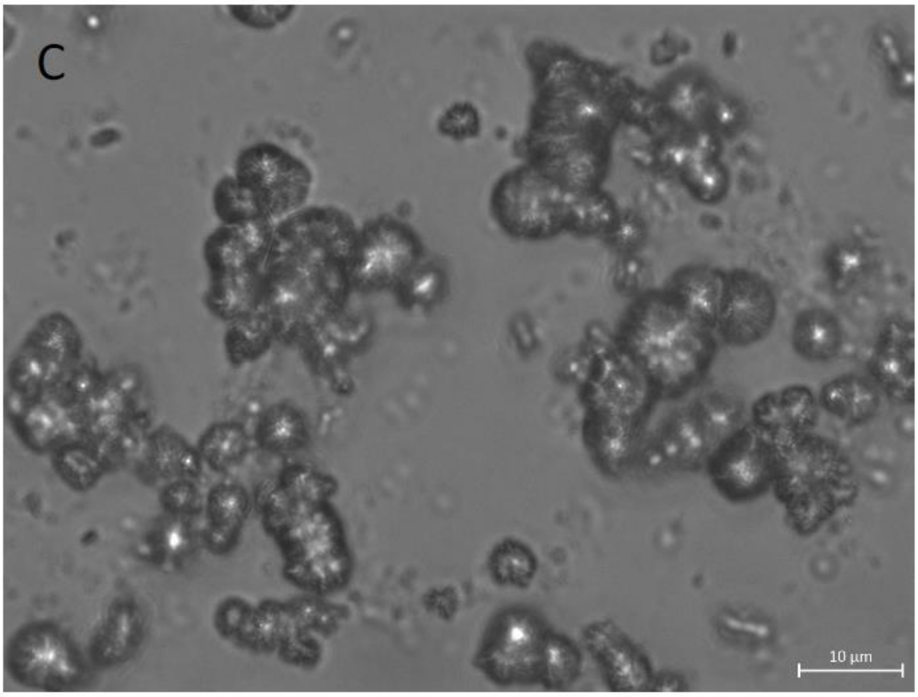


Figure S6. Optical microscopy image (100x) of microparticles obtained by aggregation of the cross-linker molecules (Cys+CDI).

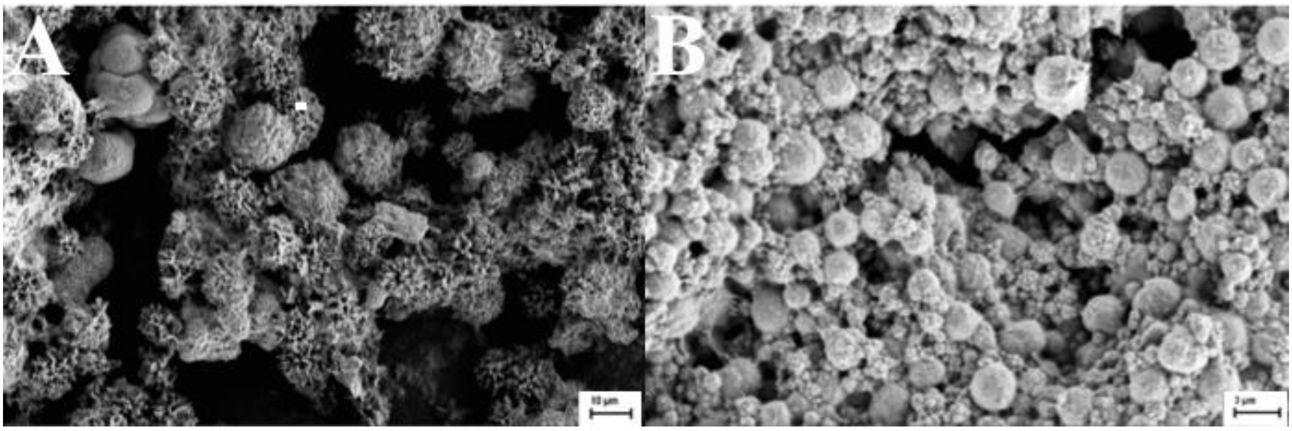


Fig. S7. SEM image of microstructures formed by : A) CL molecules (scale bar 10 µm); B) NaAlg-CL polymer (scale bar: 3 µm).

**Section S5. Determination of the size distribution of alginate microsponges**

**Confocal Laser Scanning Fluorescence Microscopy**

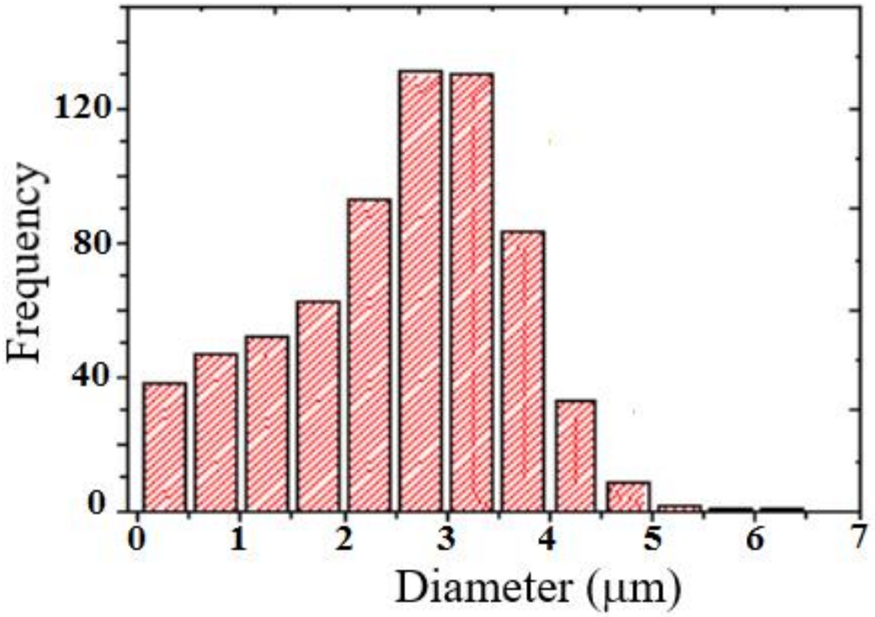


Fig. S8. Size distribution (frequency) of NaAlg PMS. The polymer was functionalised by rhodamine B (RBITC). The histograms were obtained by software analysis (ImageJ) of fluorescence confocal microscopy images.

**DoE analysis of DLS experiments**

Let us consider the PMS diameter (µm), as provided by DLS experiments, the output parameter Y, and the Cys and CDI weights (mg), and $H_2O$ volumes (µL) the input variables of the process. Output and input variables are linked by the functional relation:

$$diameter = f(Cys, CDI, H_2O)$$

To the aim we applied a $2^3$, *i.e.,* 2 values for each variable, regression model (Table S2).

Table S2. Input parameters (X) of DoE analysis

| | *Parameter* | *Unit* | *Low level (-1)* | *High level (+1)* |
|---|---|---|---|---|
| $X_1$ | Cys | mg | 20 | 30 |
| $X_2$ | CDI | mg | 20 | 30 |
| $X_3$ | $H_2O$ | µL | 200 | 300 |

A $2^3$ DoE analysis requires 8 experiments and 7 parameters. Three parameters are associated to the {Cys, CDI, $H_2O$} concentrations, and four to the {Cys · CDI, Cys · $H_2O$, CDI · $H_2O$, Cys · CDI · $H_2O$} interactions. As all experiments were carried out in duplicate,

a total of 16 data sets were considered. In the regression analysis, 'coded variables', $x_i^*$, were used. The latter can be obtained by a linear combination of the natural variables $x_i$:

$$x_i^*(x_i) = \frac{x_i - \bar{x}_i}{(x_{high} - x_{low})/2} \qquad (i = 1, \dots, 3)$$

Determining the low (-1) and high (+1) levels introduced in Table S2.

PMS diameters are therefore obtained by the DoE equation:

$$Diameter = C_0^* + C_{Cys}^* Cys^* + C_{CDI}^* CDI^* + C_{H_2O}^* H_2O^* + C_{CysCDI}^* Cys^* CDI^* + C_{CysH_2O}^* Cys^* H_2O^* + C_{CDIH_2O}^* CDI^* H_2 + C_{CysCDIH_2O}^* Cys^* CDI^* H_2O^*$$

Using 'coded variables' allow to order the selected parameters in terms of their significance in the regression analysis, in our case $\{C_{Cys}^*, C_{CDI}^*, C_{H_2O}^*, C_{CysCDI}^*, C_{CysH_2O}^*, C_{CDIH_2O}^*, C_{CysCDIH_2O}^*\}$. Coded variables are orthogonal and this makes possible to evaluate each parameter independently, allowing to dismiss separately non-significant parameters without affecting the statistical confidence of the others.

The experimental input and output parameters are reported in Table S3.

Table S3 – Values of the 'coded variables' and relative output values

| | ***Input ($X_i$) - coded*** | | | ***Output (Y)*** |
|---|---|---|---|---|
| ***order*** | ***Cys*** | ***CDI*** | ***$H_2O$*** | ***diameter (nm)*** |
| 1 | -1 | -1 | -1 | 1874 |
| 2 | +1 | -1 | -1 | 3449 |
| 3 | -1 | +1 | -1 | 7140 |
| 4 | +1 | +1 | -1 | 8635 |
| 5 | -1 | -1 | +1 | 4616 |
| 6 | +1 | -1 | +1 | 4361 |
| 7 | -1 | +1 | +1 | 4659 |
| 8 | +1 | +1 | +1 | 6572 |
| 9 | -1 | -1 | -1 | 5963 |
| 10 | +1 | -1 | -1 | 3282 |
| 11 | -1 | +1 | -1 | 5055 |
| 12 | +1 | +1 | -1 | 2877 |
| 13 | -1 | -1 | +1 | 5139 |
| 14 | +1 | -1 | +1 | 4903 |
| 15 | -1 | +1 | +1 | 7107 |
| 16 | +1 | +1 | +1 | 1929 |

The statistical significance of the regression analysis was determined by evaluating the p and T parameters. p is the probability to observe a certain value within a distribution. A selected threshold of p establishes if the value analysed can be accepted or neglected. The T parameter is associated to difference between the average values provided by the experimental and reference distribution. High values of p and low absolute values of T attest the statistical significance of the data.

The quality of the adopted DoE model is assessed by the $R^2$ parameter, representing the percent variation of the output variable Y accounted by the applied regression analysis. A better parameter, that considers the number of regressor variables (p) with respect to the number of experimental data (n) is $R^2_{Adj}$:

$$R^2_{Adj} = 1 - (1 - R^2)\frac{p}{n - p - 1}$$

The results of the applied regression analysis using a statistical threshold $\alpha$= 0.25 for the chosen regressor variables are reported in Table S4.

Table S4 – Statistical assessment of the DoE model applied for the determination of the average diameter of NaAlg microsponges

| | ***Regressor*** | ***Coefficients*** | | ***p*** | ***T*** | ***Order of significance*** |
|---|---|---|---|---|---|---|
| | *Constant* | $C_0^*$ | - 10925 | | - 2,59 | |
| | *Cys* | $C_{Cys}^*$ | - | > 0,25 | | |
| | *CDI* | $C_{CDI}^*$ | - | > 0,25 | | |
| | *$H_2O$* | $C_{H_2O}^*$ | 97,5 | 0,011 | 3,05 | 3 |
| | *Cys · CDI* | $C_{CysCDI}^*$ | 24,90 | 0,002 | 3,91 | 2 |
| | *Cys · $H_2O$* | $C_{CysH_2O}^*$ | - 2,669 | 0,002 | - 4,12 | 1 |
| | *CDI · $H_2O$* | $C_{CDIH_2O}^*$ | - 1,232 | 0,084 | - 1,90 | 4 |
| | *Cys · CDI ·$H_2O$* | $C_{CysCDIH_2O}^*$ | - | > 0,25 | | |
| $\boldsymbol{R^2}$ | | 80,9 % | | | | |
| $\boldsymbol{R^2_{Adj}}$ | | 74,0 % | | | | |

The values obtained for $R^2$ (80.9%) and $R^2_{Adj}$ (74.0%) make us confident on the applied DoE analysis of the experimental data.

The Pareto hystograms [1] (Figure S9) report the number of data with statistical significance (standardized effects) larger than 0.25 for some of the analysed regressor variables. From this analysis, the most relevant factor is the {Cys · $H_2O$} interaction,

followed, with decreasing effect, by {Cys · CDI}, {$H_2O$} and {CDI · $H_2O$}. It is also possible evaluate the contribution of each factor to the output with respect to the normal distribution (Figure S9B). Coded variables showing negative values with respect to such distribution decrease the statistical significance of the output on going from the low to the high level, while positive values increase the quality of the answer. The data reported in Figure S9 show that the {Cys · $H_2O$} and {CDI · $H_2O$} regressors cause a decrease in the statistical significance of the output, while the {$H_2O$} and {Cys · CDI} factors increase such parameter.

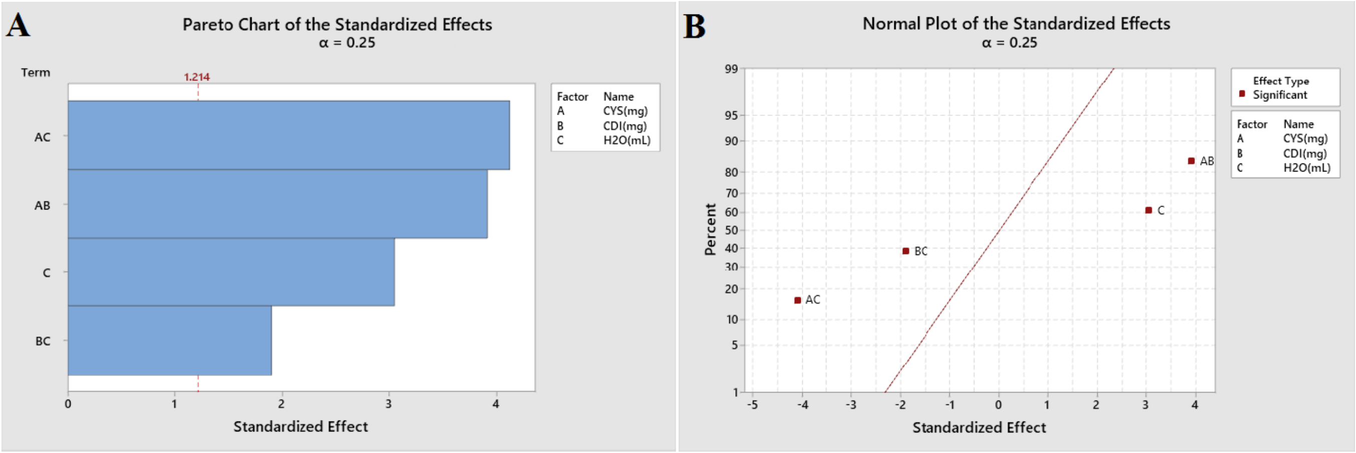


Figure S9. A) Standardized effect of the analysed coded variable (Pareto histogram); B) Deviation of the standardized effect from the normal distribution (dotted line).

The adopted statistical model (DoE), applied to the DLS experimental data sets to determine the diameter of the NaAlg PMS, provided the following equation:

$$diameter = -10925 + 97.5\, H_2O + 24.90\, Cys \cdot CDI - 2.669\, Cys \cdot H_2O - 1.232\, CDI \cdot H_2O \qquad (3.7)$$


1. Montgomery D.C., *Design and Analysis of Experiments*, New York: Wiley, 2005


Table S5. Distribution analysis of DLS data for the formulations (DoE items)

| DoE item | Peaks | Diam (nm) | % Intensity | Width (nm) | Z-average[1] (d.nm) | PdI[2] |
|---|---|---|---|---|---|---|
| **DoE 1** | 2 | 7390<br>2213 | 53.4<br>46.6 | 1092<br>451.2 | 3750 | 0.352 |
| | 1 | 4378 | 100 | 1439 | 3744 | 0.155 |
| | 2 | 5055<br>1032 | 96.5<br>3.5 | 1733<br>194.8 | 4160 | 0.172 |
| | 2 | 2369<br>6503 | 56.9<br>43.1 | 804.3<br>1442 | 3003 | 0.292 |

| | | | | | | |
|---|---|---|---|---|---|---|
| **DoE 2** | 2 | 1928<br>7341 | 51.6<br>48.4 | 412.8<br>1112 | 3192 | 0.347 |
| | 1 | 4659 | 100 | 2154 | 3655 | 0.245 |
| **DoE 3** | 2 | 7571<br>1895 | 82.9<br>17.1 | 997.8<br>346.4 | 5609 | 0.34 |
| | 2 | 7673<br>736.5 | 91.1<br>8.9 | 945.9<br>88.0 | 6020 | 0.373 |
| | 1 | 2877 | 100 | 502.7 | 2704 | 0.126 |
| **DoE 4** | 2 | 7184<br>1754 | 88.7<br>11.3 | 1168<br>303.0 | 5681 | 0.27 |
| | 2 | 5989<br>864.1 | 93.4<br>6.6 | 1625<br>230.3 | 4626 | 0.264 |
| | 1 | 1929 | 100 | 248.0 | 1779 | 0.071 |
| **DoE 5** | 2 | 7960<br>2232 | 55.9<br>44.1 | 782.5<br>359.6 | 4367 | 0.412 |
| | 1 | 6082 | 100 | 1405 | 5341 | 0.209 |
| | 1 | 3449 | 100 | 519.5 | 3197 | 0.08 |
| **DoE 6** | 1 | 6046 | 100 | 1557 | 5064 | 0.205 |
| | 1 | 5095 | 100 | 1809 | 4286 | 0.191 |
| | 1 | 3282 | 100 | 452.0 | 3278 | 0.211 |
| **DoE 7** | 2 | 6031<br>1833 | 94.5<br>5.5 | 1645<br>324.5 | 5040 | 0.204 |
| | 1 | 5460 | 100 | 1595 | 4586 | 0.155 |
| | 1 | 5963 | 100 | 1490 | 5614 | 0.117 |
| **DoE 8** | 2 | 6927<br>1820 | 61.6<br>38.4 | 1273<br>409.7 | 3367 | 0.398 |
| | 2 | 6099<br>2198 | 71.2<br>28.8 | 1691<br>550.1 | 3461 | 0.366 |
| | 1 | 4903 | 100 | 1335 | 4444 | 0.084 |
| **DoE 9** | 1 | 6920 | 100 | 1237 | 6671 | 0.186 |
| | 1 | 8281 | 100 | 549.9 | 7993 | 0.369 |
| | 1 | 6572 | 100 | 1256 | 6516 | 0.073 |
| **DoE 10** | 1 | 6719 | 100 | 1557 | 6435 | 0.201 |
| | 2 | 8440<br>775.4 | 93.8<br>6.2 | 437.8<br>55.9 | 8244 | 0.256 |
| | 1 | 7140 | 100 | 1172 | 7056 | 0.166 |
| **DoE 11** | 2 | 8098<br>2024 | 69.6<br>30.4 | 710.6<br>298.7 | 5415 | 0.398 |
| | 1 | 7693 | 100 | 936.5 | 7732 | 0.111 |
| | 1 | 5139 | 100 | 934.0 | 5165 | 0.185 |
| **DoE 12** | 1 | 5079 | 100 | 1249 | 4677 | 0.149 |
| | 2 | 6670<br>3224 | 66.6<br>33.4 | 1486<br>614.3 | 5014 | 0.214 |
| | 1 | 4616 | 100 | 810.4 | 4764 | 0.473 |
| **DoE 13** | 1 | 6982 | 100 | 1380 | 6920 | 0.15 |
| | 2 | 8307<br>1602 | 91.4<br>8.6 | 528.2<br>138.8 | 7741 | 0.287 |
| | 1 | 7107 | 100 | 1176 | 6867 | 0.218 |
| **DoE 14** | 1 | 3762 | 100 | 696.9 | 3544 | 0.055 |
| | 2 | 7964<br>1467 | 83.3<br>16.7 | 780.6<br>226.9 | 5470 | 0.482 |
| | 1 | 1874 | 100 | 249.8 | 1910 | 0.628 |
| | 2 | 8319 | 81.6 | 522.4 | 7238 | 0.398 |

| | | | | | | |
|---|---|---|---|---|---|---|
| **DoE 15** | | 3243 | 18.4 | 357.4 | | |
| | 1 | 7550 | 100 | 952.8 | 7351 | 0.186 |
| **DoE 16** | 2 | 1350 | 69.5 | 228.2 | 6044 | 0.314 |
| | | 5560 | 30.5 | | | |
| | 1 | 7260 | 100 | 1345 | 7187 | 0.241 |

[1] $Z - average = \frac{\sum_i S_i}{\sum_i (S_i/D_i)}$, where $S_i$ and $D_i$ are the scattered intensity and the diameter of the particle i, respectively.

[2] PdI = Polydispersity index.

## Section S6. CIGB814 loading to NaAlg PMS

Table S6. UV-Vis Absorption of supernatant solutions of CIGB814 (0.5 mg/ml) and NaAlg PMS (4 mg) at different times.

| | *Incubation time (h)* | *Abs (280 nm) ± SD* | *CIGB814 concentration (supernatant) (mg/mL) ± SD* | *CIGB814 loading (%) ± SD* |
|---|---|---|---|---|
| *NaAlg PMS (4 mg)* | - | 0.031 ± 0.002 | - | - |
| *CIGB814 0.5 mg/mL* | - | 0.272 ± 0.003 | 0.552 ± 0.006 | 0 |
| *NaAlg2 PMS + CIGB814 (supernatant)* | 0 | 0.215 ± 0.003 | 0.436 ± 0.006 | 21 ± 1 |
| | 0.5 | 0.222 ± 0.003 | 0.387 ± 0.006 | 30 ± 1 |
| | 1 | 0.225 ± 0.001 | 0.394 ± 0.003 | 28.5 ± 0.5 |
| | 2 | 0.215 ± 0.007 | 0.37 ± 0.01 | 32 ± 3 |
| | 3 | 0.208 ± 0.007 | 0.360 ± 0.001 | 34.82 ± 0.02 |
| | 4 | 0.216 ± 0.002 | 0.375 ± 0.004 | 31.9 ± 0.7 |

Table S7. UV-Vis Absorption of supernatant solutions of CIGB814 (0.5 mg/ml) and NaAlg PMS (8 mg) at different times.

| | *Incubation time (h)* | *Abs (280 nm) ± SD* | *CIGB814 Concentration (supernatant) (mg/mL) ± SD* | *CIGB814 loading (%) ± SD* |
|---|---|---|---|---|
| *Microsponges (8 mg)* | - | 0.06 ± 0.02 | - | - |
| *CIGB814 0.5 mg/mL* | - | 0.254 ± 0.002 | 0.514 ± 0.003 | 0 |
| *Microsponges+ CIGB814* | 0 | 0.24 ± 0.01 | 0.48 ± 0.02 | 6 ± 4 |
| | 0.5 | 0.144 ± 0.004 | 0.160 ± 0.007 | 69 ± 1 |
| | 1 | 0.16 ± 0.02 | 0.19 ± 0.04 | 61 ± 7 |
| | 2 | 0.159 ± 0.003 | 0.190 ± 0.006 | 63 ± 1 |
| | 3 | 0.15 ± 0.02 | 0.17 ± 0.05 | 66 ± 9 |
| | 4 | 0.16 ± 0.02 | 0.18 ± 0.04 | 64 ± 7 |

Table S8. Fluorescence emission intensities and fractional loading (%) of supernatant solutions of CIGB814 (0.5 mg/ml) and NaAlg PMS (4 mg) at different times.

| | *Incubation time (h)* | *Fluorescence intensity ($\lambda_{em}$=307 nm) ± SD* | *CIGB814 loading (%) ± SD* |
|---|---|---|---|
| *NaAlg PMS (4 mg)* | - | 41769 ± 1067 | - |
| *CIGB814 0.5 mg/mL* | - | 2.49 ± 0.01 E+06 | 0 |
| *NaAlg PMS + CIGB814* | 0 | 2.22 ± 0.11 E+06 | 12 ± 5 |
| | 0.5 | 1.73 ± 0.02 E+06 | 32.1 ± 0.9 |
| | 1 | 1.64 ± 0.04 E+06 | 36 ± 2 |
| | 2 | 1.58 ± 0.01 E+06 | 38.3 ± 0.4 |
| | 3 | 1.57 ± 0.07 E+06 | 38 ± 3 |
| | 4 | 1.60 ± 0.01 E+06 | 37 ± 0.4 |

Table S9. Fluorescence emission intensities and fractional loading (%) of supernatant solutions of CIGB814 (0.5 mg/ml) and NaAlg PMS (8 mg) at different times.

| | *Incubation time (h)* | *Fluorescence intensity (307 nm) ± SD* | *CIGB814 loading (%) ± SD* |
|---|---|---|---|
| *NaAlg PMS (8 mg)* | - | 16789 ± 1090 | - |
| *CIGB814 (0.5 mg/ml)* | - | 2.49 ± 0.01 E+06 | 0 |
| *NaAlg PMS + CIGB814* | 0 | 1.57 ± 0.26 E+06 | 37 ± 10 |
| | 0.5 | 7.44 ± 0.25 E+05 | 70 ± 1 |
| | 1 | 7.02 ± 0.35 E+05 | 72 ± 1 |
| | 2 | 6.08 ± 0.18 E+05 | 75.5 ± 0.7 |
| | 3 | 5.72 ± 0.43 E+05 | 77 ± 2 |
| | 4 | 5.52 ± 0.14 E+05 | 77.8 ± 0.6 |

**Section S7. CIGB814 release from NaAlg2 PMS in PBS pH=7.4**

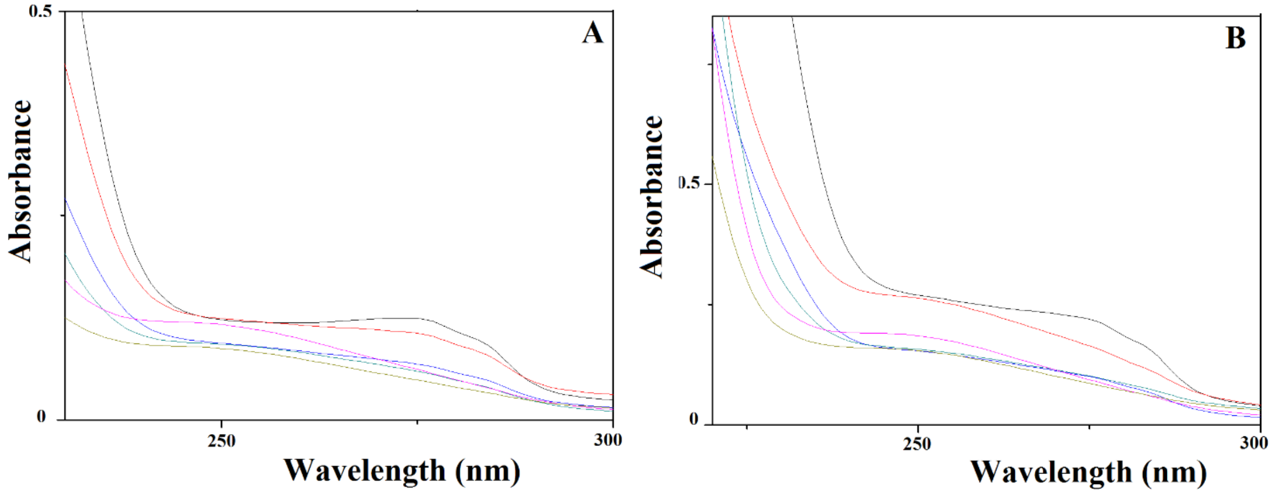


Figure S10. UV-Vis absorption spectra of supernatant solutions of CIGB814/NaAlg PMS for increasing incubation times in PBS (pH=7.4). CIGB814: 0.5 mg/mL; NaAlg PMS: A) 4 mg/mL; B) 8 mg/mL. Black: 15 min; Red: 1 h; Blue: 2 h; Emerald: 24 h; Purple: 92 h; Olive:116 h.

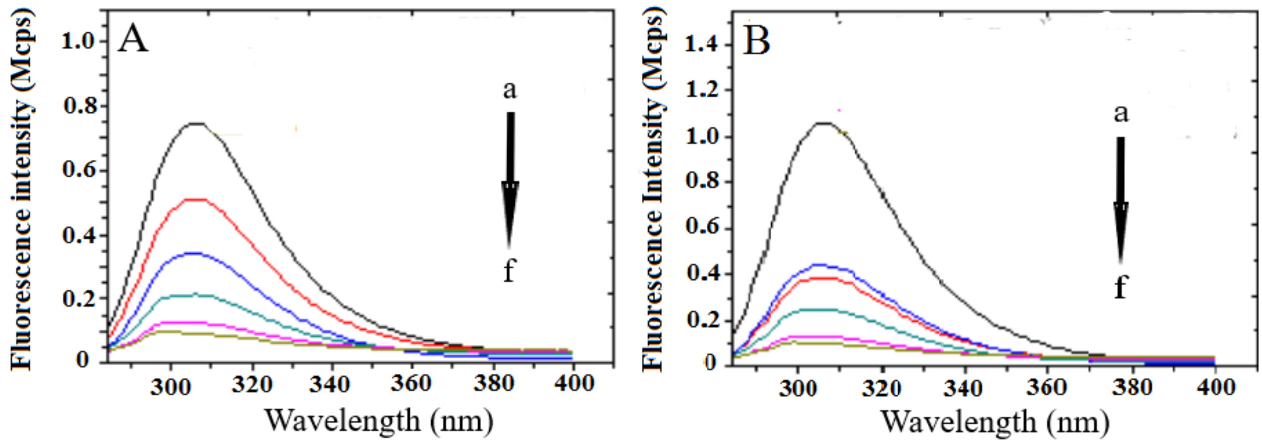


Figure S11. Fluorescence emission spectra (Mcps=106 count per second) of CIGB814/NaAlg PMS supernatant solutions at increasing incubation times in PBS (pH=7.4). A) CIGB814: 0.19 mg/mL; NaAlg PMS: 4 mg/mL; B) CIGB814: 0.37 mg/mL; NaAlg PMS: 8 mg/mL (C). a) 15 min; b) 1h; c) 2h; d) 24h; e) 92h; f) 116h.

Table S10. Fluorescence emission data and fractional release (%) of supernatant solutions of NaAlg PMS (4 mg)/CIGB814 (0.5 mg/ml) in PBS pH 7.4 at different incubation times.

| | *Incubation time (h)* | *Fluorescence Intensity (cps) ($\lambda_{em}$=307 nm) ± SD* | *CIGB814 release (%) ± SD* |
|---|---|---|---|
| *NaAlg PMS (4 mg)* | 0.25 | 25419 ± 1091 | - |
| | 1 | 38244 ± 1089 | - |
| | 2 | 29978 ± 1090 | - |
| | 24 | 30285 ± 1007 | - |
| | 92 | 46595 ± 1091 | - |
| | 116 | 51367 ± 1080 | - |
| *Loaded CIGB814* | - | 1.39 ± 0.41 E+06 | 100 |
| *NaAlg + CIGB814 (PBS, pH 7.4)* | 0.15 | 7.48 ± 0.71 E+05 | 52 ± 5 |
| | 1 | 5.09 ± 0.13 E+05 | 59.8 ± 0.9 |
| | 2 | 3.40 ± 0.13 E+05 | 71.0 ± 0.9 |
| | 24 | 2.13 ± 0.09 E+05 | 73.0 ± 0.9 |
| | 92 | 1.24 ± 0.05 E+05 | 73.0 ± 0.9 |
| | 116 | 9.29 ± 0.44 E+04 | 73.2 ± 0.9 |

Table S11. Fluorescence emission data and fractional release (%) of supernatant solutions of NaAlg PMS (8 mg)/CIGB814 (0.5 mg/ml) in PBS pH 7.4 at different incubation times.

| | *Incubation time (h)* | *Fluorescence Intensity (cps) ($\lambda_{em}$=307 nm) ± SD ± SD* | *CIGB814 release (%) ± SD* |
|---|---|---|---|
| *NaAlg PMS (8 mg)* | 0.25 | 37854 ± 1050 | - |
| | 1 | 65023 ± 1009 | - |
| | 2 | 27522 ± 1032 | - |
| | 24 | 33132 ± 1006 | - |
| | 92 | 39315 ± 1090 | - |
| | 116 | 51220 ± 1023 | - |
| *CIGB814 loaded* | - | 1.98 ± 0.41 E+06 | 100 |
| *NaAlg PMS + CIGB814 + PBS (pH 7.4)* | 0.25 | 1.06 ± 0.09 E+06 | 51 ± 5 |
| | 1 | 6.62 ± 0.66 E+05 | 56 ± 3 |
| | 2 | 4.37 ± 0.28 E+05 | 66 ± 3 |
| | 24 | 2.50 ± 0.08 E+05 | 67 ± 3 |
| | 92 | 1.29 ± 0.09 E+05 | 67 ± 3 |
| | 116 | 9.79 ± 0.72 E+04 | 67 ± 3 |
